# Dental Implant Placement with Simultaneous Anterior Maxillary Reconstruction with Block and Particulate Fresh Frozen Allograft Bone: A Case Report with 24-Month Follow-Up Data

**DOI:** 10.1155/2017/1565973

**Published:** 2017-02-16

**Authors:** J. S. Vieira, E. M. Brandão-Filho, F. R. Deliberador, J. C. Zielak, A. F. Giovanini, T. M. Deliberador

**Affiliations:** ^1^Graduate Program in Dentistry, Positivo University, Curitiba, PR, Brazil; ^2^Private Practice, Balneário Camboriú, SC, Brazil

## Abstract

Fresh frozen allograft bone is routinely used in orthopedic surgery for the reconstruction of large bone defects, and its use in oral and maxillofacial surgery is increasing. The purpose of this case was to demonstrate the installation of dental implants and the use of fresh frozen bone for reconstruction of anterior maxilla in the same surgery. This case report presents the insertion of dental implants followed immediately by a placement of fresh frozen allograft in block and particle for a reconstruction of atrophic anterior maxillary in the same surgery. Ten months subsequent to this procedure, provisional fixed prosthesis was installed on the implants. Four months later (postoperative month 14), the final fixed prosthesis was installed and the clinical success was observed. The insertion of dental implants followed immediately by a placement of fresh frozen allograft is a safe and efficient process that results in the successful return of dental function and aesthetic rehabilitation for the patient.

## 1. Introduction

Bone grafting techniques are widely used in the restoration of atrophic maxillary bone prior to placement of dental implants. Atrophy of the maxillary bone is caused by trauma, oncologic diseases, oral infections, congenital absence of teeth, or the tridimensional alveolar ridge resorption process subsequent to following dental extractions [1].

The rehabilitation of large bone defects can be achieved with various types of grafting materials and may be natural or synthetic. The use of autologous grafts exhibits the highest success rate, and autogenous block grafts are considered the gold standard because their osteogenic, osteoinductive, and osteoconductive properties maximize the success of graft incorporation [2, 3]. Nevertheless, the use of autogenous bone grafts also presents considerable drawbacks, including high morbidity at the donor site, limited quantity of bone, unpredictable quality of bone, increased blood loss, increased operative time, and infection at the donor site [4].

Although autogenous bone grafts remain the preferred reconstructive method, there has been an increased use of fresh frozen bone allografts in oral and maxillofacial surgery [5]. The advantages of using fresh frozen bone allografts include decreased operative trauma for the patient, a nearly unlimited supply of reconstructive material, decreased blood loss, absence of donor-site morbidity, decreased operative time, and convenience for the surgeon [6].

In 2009, Contar and colleagues [7] conducted a study of 15 patients with atrophy of the maxillary ridge who required bone block grafts prior to implant placement. These patients underwent maxillary reconstructions performed with block grafts comprising fresh frozen chips harvested from allogeneic tibial bone. They concluded that block fresh frozen allografts could be used successfully in the treatment of maxillary ridge defects.

In 2014, Pereira and colleagues [1] performed a prospective cohort study aimed at clinically evaluating the extent of resorption over time of corticocancellous fresh frozen allograft bone blocks used in the reconstruction of severe maxillary atrophy. They found that the use of fresh frozen allograft bone harvested from the iliac crest is a suitable alternative in atrophic maxillary reconstruction and exhibited a low resorption rate at 5 months, which allowed for proper stability of dental implants prior to fixed prosthetic rehabilitation.

The purpose of this case was to demonstrate the installation of dental implants and the use of fresh frozen bone for reconstruction of anterior maxilla in the same surgery.

## 2. Case Presentation

**Figure 1:**
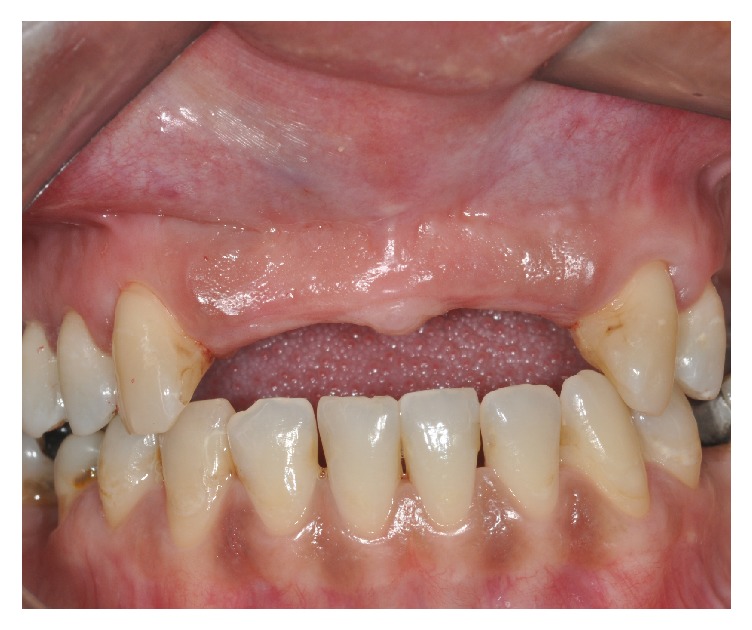
Maxillary: preoperative view.

Patient CG, a 38-year-old woman who was currently wearing an upper anterior partial prosthesis, sought treatment for oral rehabilitation through osseointegrated implants. The patient presented with aesthetic complaints, and there was no relevant medical history. Clinical examination showed the absence of the anterior maxillary teeth (Figure 1).

Preoperative imaging with computed tomography revealed severe atrophy of the anterior maxilla extending 2.8 mm in the vertical direction and 13 mm in the horizontal direction. The proposed treatment involved installation of two implants (3.3 mm × 11.5 mm) in the lateral incisor areas and reconstruction of the anterior maxilla through block grafts and particulate bone consisting of allografts during the same surgical session.

**Figure 2:**
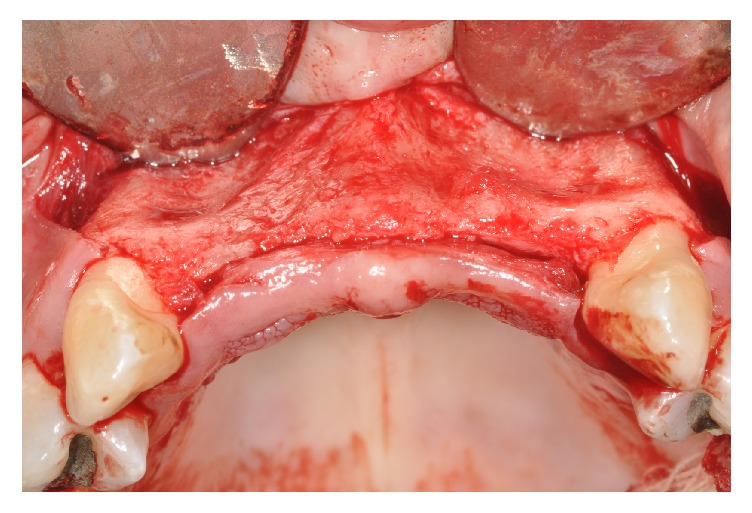
The total-thick flap raised.

The patient received amoxicillin 2000 mg for antibiotic prophylaxis and 8 mg of dexamethasone one hour prior to surgery. Following intra- and extraoral antisepsis and local anesthesia (4% articaine with 1 : 100,000 epinephrine; DFL, Rio de Janeiro, Brazil), a straight incision with a size 15 scalpel blade was executed along the maxillary alveolar ridge from the mesial side of tooth 13 to the mesial side of tooth 23. Following that, a perpendicular releasing incision was performed on the distal sides of teeth 13 and 23. Using a Molt elevator, the full-thickness flap was reflected toward the base of the vestibule to expose the bone remnant (Figure 2).

**Figure 3:**
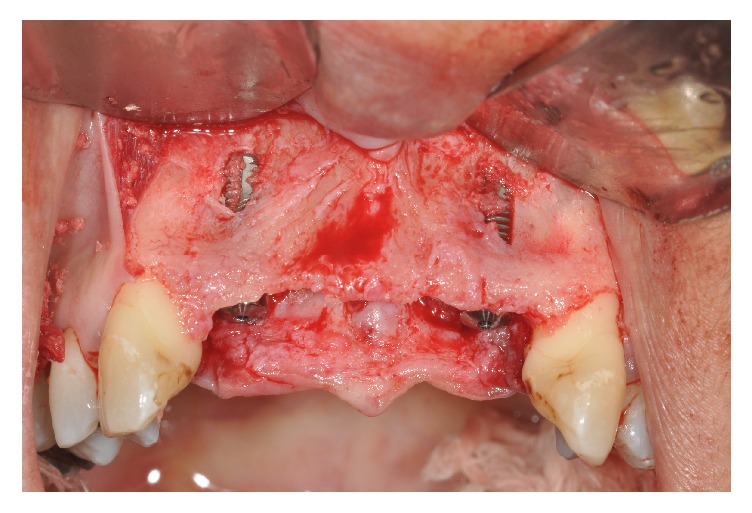
The implants installed.

Implant sites were prepared with drills and the implants installed using a palatal approach and locked with 15N. Due to the severely attenuated thickness of the maxilla, fenestration occurred on the vestibular side (Figure 3).

**Figure 4:**
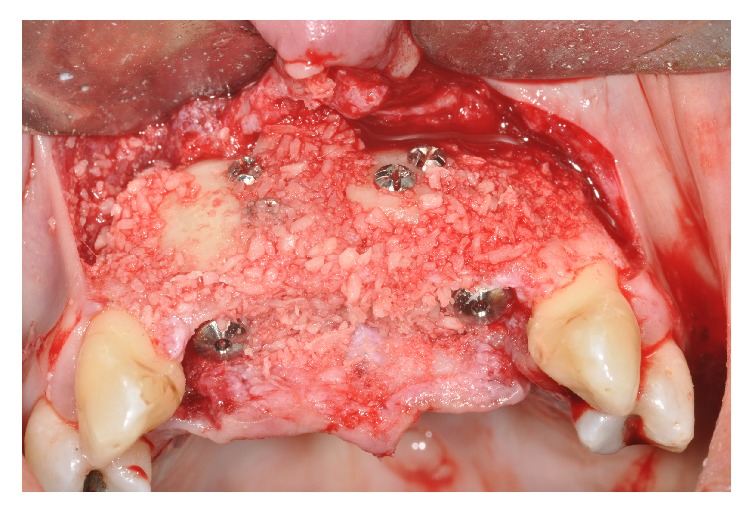
Bone blocks fixed with miniscrews and particulate bone.

Following careful preparation of the site, which included decortication of the maxilla to enhance marrow space bleeding, the blocks were perfectly adapted to the maxillary wall without any gap. Fresh frozen bone was obtained from Santa Casa, São Paulo, SP, Brazil, bone bank. Fixation with miniscrews (Systhex, Curitiba, Brazil) stabilized the prepared blocks at the recipient site. To prevent micromovement of the graft, miniscrews were placed through the central portion of the blocks and came to rest in the palatal portion of the maxilla. Particle bone was placed around the blocks to eliminate gaps (Figure 4).

**Figure 5:**
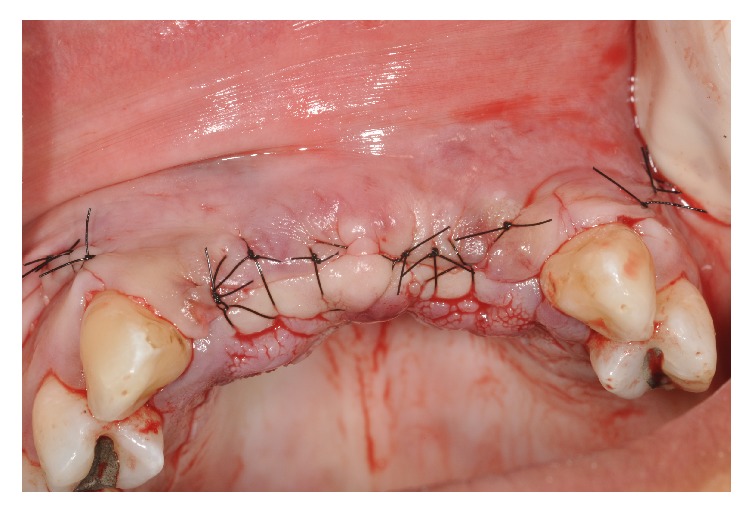
Suture.

The flap was repositioned without tension, and nylon sutures (number 5.0; Ethicon, Somerville, NJ) were used for closure (Figure 5).

Following surgery, the patient received an analgesic (paracetamol, 750 mg every 6 hours for 4 days), an antibiotic (amoxicillin, 500 mg every 8 hours for 7 days), chemical plaque control (0.12% chlorhexidine gluconate rinse every 12 hours for 14 days), and anti-inflammatory medication (ibuprofen, 600 mg every 8 hours for 4 days). The suture was removed on postoperative day 14. The patient was maintained under professional supervision to ensure proper oral hygiene.

**Figure 6:**
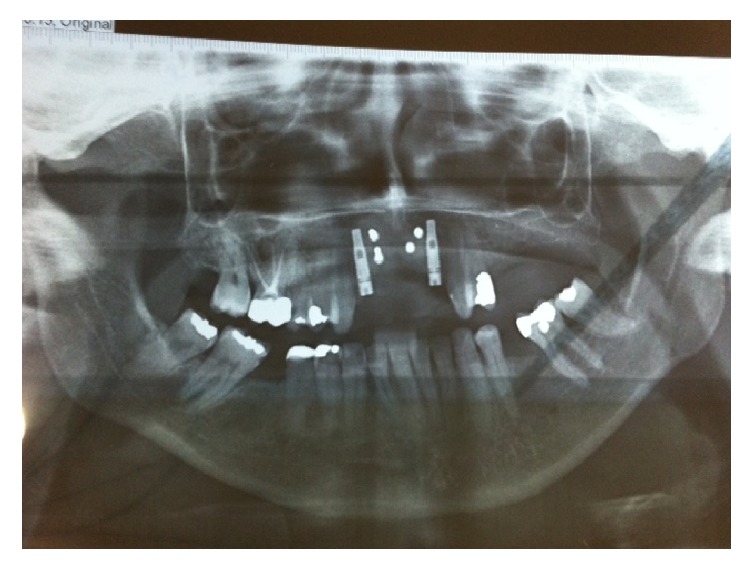
Panoramic radiograph after 10 months postoperatively.

In the tenth postoperative month, a panoramic radiograph was obtained to evaluate the graft and osseointegration of the implant (Figure 6).

**Figure 7:**
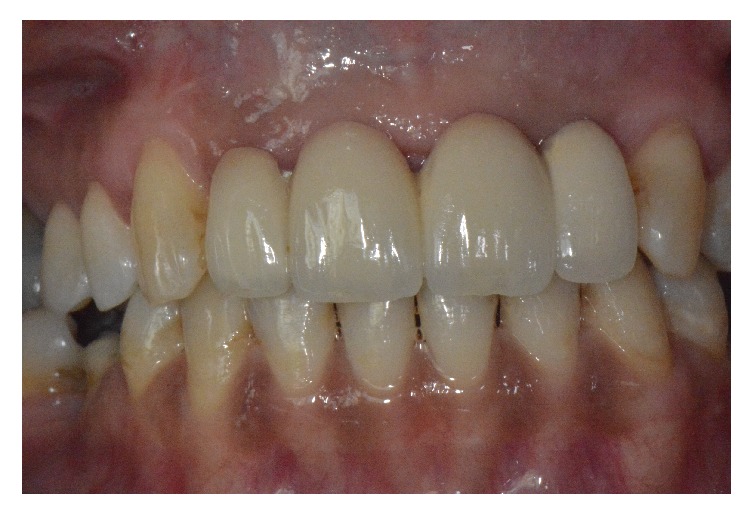
Ceramic prosthesis.

At that time, a provisional fixed prosthesis was installed on the implants. Four months later (postoperative month 14), the final fixed prosthesis was installed. The patient was followed closely for a total of 24 months after the surgery (Figure 7).

## 3. Discussion

In the present case, we have demonstrated that the insertion of dental implants followed immediately by a placement of fresh frozen allograft is a safe and efficient process that results in the successful return of dental function and aesthetic rehabilitation for the patient.

Concurrent bone grafting and placement of dental implants effectively minimizes treatment time. The highly satisfactory results we report here are in accordance with the case studies presented by other authors [1, 7, 8].

Demineralized freeze dried bone allografts have proven to be successful in terms of integration with the host bone due to their osteoinductive potential [9, 10]. However, the allograft bone used in our study was only fresh and frozen and did not possess osteoinductive properties, only osteoconductive potential. In addition, FFB allografts offer several benefits when compared to autologous grafts, namely, reductions in morbidity, discomfort, and surgical time [6]. One disadvantage of allografts is the risk of infectious disease transmission [6]. However, with the adherence to standard safety protocols by bone banks, the risk of viral transmission by unprocessed deep-frozen, nonirradiated grafts from screened donors is extremely unlikely at the current time [11].

In 2013, Spin-Neto [12] and colleagues performed a histological analysis of fresh frozen onlay bone allografts, compared with autografts used in maxillary reconstruction prior to dental implant placement [12]. They concluded that human fresh frozen bone block allografts demonstrated clinical compatibility for grafting procedures but were associated with a slow remodeling process. In light of this, we waited for 10 months to reopen and apply a fixed prosthesis.

Another recent study evaluated fresh frozen human bone allografts used in vertical ridge augmentation both clinically and with computed tomography in order to assess resultant bone formation and graft resorption [13]. The researchers concluded that fresh frozen allografts promote satisfactory vertical bone formation as well as low resorption rates, good density, and implant stability. In our case study, bone resorption was not clinically observed, and ridge thickness was maintained throughout the 24-month follow-up period.

## References

[1] Pereira E., Messias A., Dias R., Judas F., Salvoni A., Guerra F. (2015). Horizontal resorption of fresh-frozen corticocancellous bone blocks in the reconstruction of the atrophic maxilla at 5 months. *Clinical Implant Dentistry and Related Research*.

[2] Zetola A. L., Verbicaro T., Littieri S., Larson R., Giovanini A. F., Deliberador T. M. (2014). Recombinant human bone morphogenetic protein type 2 in the reconstruction of atrophic maxilla: case report with long-term follow-up. *Journal of Indian Society of Periodontology*.

[3] Albrektsson T., Johansson C. (2001). Osteoinduction, osteoconduction and osseointegration. *European Spine Journal*.

[4] Shand J. M., Heggie A. A. C., Holmes A. D., Holmes W. (2002). Allogeneic bone grafting of calvarial defects: an experimental study in the rabbit. *International Journal of Oral and Maxillofacial Surgery*.

[5] Carinci F., Guidi R., Franco M. (2009). Implants inserted in fresh-frozen bone: a retrospective analysis of 88 implants loaded 4 months after insertion. *Quintessence International*.

[6] Perrott D. H., Smith R. A., Kaban L. B. (1992). The use of fresh frozen allogeneic bone for maxillary and mandibular reconstruction. *International Journal of Oral and Maxillofacial Surgery*.

[7] Contar C. M. M., Sarot J. R., Bordini J., Galvão G. H., Nicolau G. V., Machado M. A. N. (2009). Maxillary ridge augmentation with fresh-frozen bone allografts. *Journal of Oral and Maxillofacial Surgery*.

[8] Wallowy P., Dorow A. (2012). Lateral augmentation of the maxilla and mandible using framework technique with allogeneic bone grafts. *Journal of Oral Implantology*.

[9] Dallari D., Fini M., Stagni C. (2006). In vivo study on the healing of bone defects treated with bone marrow stromal cells, platelet-rich plasma, and freeze-dried bone allografts, alone and in combination. *Journal of Orthopaedic Research*.

[10] Myerson M. S., Neufeld S. K., Uribe J. (2005). Fresh-frozen structural allografts in the foot and ankle. *Journal of Bone and Joint Surgery*.

[11] Albert A., Leemrijse T., Druez V., Delloye C., Cornu O. (2006). Are bone autografts still necessary in 2006? A three-year retrospective study of bone grafting. *Acta Orthopaedica Belgica*.

[12] Spin-Neto R., Landazuri Del Barrio R. A., Pereira L. A. V. D., Marcantonio R. A. C., Marcantonio E., Marcantonio E. (2013). Clinical similarities and histological diversity comparing fresh frozen onlay bone blocks allografts and autografts in human maxillary reconstruction. *Clinical Implant Dentistry and Related Research*.

[13] Macedo L. G. S., Mazzucchelli-Cosmo L. A., Macedo N. L., Monteiro A. S. F., Sendyk W. R. (2012). Fresh-frozen human bone allograft in vertical ridge augmentation: clinical and tomographic evaluation of bone formation and resorption. *Cell and Tissue Banking*.

